# Independent Evolutionary Lineages in a Globular Cactus Species Complex Reveals Hidden Diversity in a Central Chile Biodiversity Hotspot

**DOI:** 10.3390/genes13020240

**Published:** 2022-01-27

**Authors:** Heidy M. Villalobos-Barrantes, Beatriz M. Meriño, Helmut E. Walter, Pablo C. Guerrero

**Affiliations:** 1 Departamento de Botánica, Facultad de Ciencias Naturales y Oceanográficas, Universidad de Concepción, Concepción 4030000, Chile; hemaviba@gmail.com (H.M.V.-B.); b.vergara.m@gmail.com (B.M.M.); 2 Escuela de Química, Universidad de Costa Rica, San José 11501-2060, Costa Rica; 3 Centro de Investigación en Biología Celular y Molecular, Universidad de Costa Rica, San José 11501-2060, Costa Rica; 4 Institute of Ecology and Biodiversity (IEB), Concepción 4030000, Chile; 5 The EXSIS Project: Cactaceae Ex-Situ & In-Situ Conservation, 31860 Emmerthal, Germany; hw582133@gmail.com; 6 Millennium Institute Biodiversity of Antarctic and Sub-Antarctic Ecosystems (BASE), Santiago 7800003, Chile

**Keywords:** endemism, Cactaceae, Neotropical biodiversity, Mediterranean biome, Central Chile

## Abstract

Unraveling the processes involved in the origin of a substantial fraction of biodiversity can be a particularly difficult task in groups of similar, and often convergent, morphologies. The genus *Eriosyce* (Cactaceae) might present a greater specific diversity since much of its species richness might be hidden in morphological species complexes. The aim of this study was to investigate species delimitation using the molecular data of the globose cacti “*E.* *curvispina*”, which harbor several populations of unclear evolutionary relationships. We ran phylogenetic inferences on 87 taxa of *Eriosyce*, including nine *E. curvispina* populations, and by analyzing three plastid noncoding introns, one plastid and one nuclear gene. Additionally, we developed 12 new pairs of nuclear microsatellites to evaluate the population-level genetic structure. We identified four groups that originated in independent cladogenetic events occurring at different temporal depths; these groups presented high genetic diversity, and their populations were genetically structured. These results suggest a complex evolutionary history in the origin of globular cacti, with independent speciation events occurring at different time spans. This cryptic richness is underestimated in the Mediterranean flora of central Chile, and thus unique evolutionary diversity could be overlooked in conservation and management actions.

## 1. Introduction

The planet’s biodiversity is declining at unprecedented rates [1]. Mediterranean regions are among the most unique and threatened ecosystems, characterized by mild, wet winters and very dry, long, hot summers [2,3], and harboring 20 percent (%) of species even when having a surface less than 5% the of total global landmass [4]. The Mediterranean area in Central Chile is recognized by the high number of endemic species, many of which are heavily threatened [4,5]. Since the Chilean economy has long depended on its natural capital for economic development, there has been a severe impact on even its most biodiverse ecosystems, from agriculture, to housing, mining and deforestation [6]. Although knowledge of the biodiversity of Central Chile has increased in recent decades, there are still important gaps, especially in non-charismatic animal groups and non-woody plants. Moreover, in the last decade, a large number of angiosperms belonging to different families, such as Alstromeriaceae [7,8], Amarillidaceae [9,10], Brassicaceae [11], Orchidaceae [12] and Cactaceae [13,14], have been described.

The underestimation of diversity may be due, in part, to the occurrence of species that are sufficiently distinct based on molecular characterizations, but which have been classified as a single nominal species because they are at least superficially morphologically indistinguishable [15]. Estimates of species diversity and levels of endemism should consider the possible occurrence of cryptic species that could go unnoticed or be synonymized when relying only on morphological approaches in their classification, because they do not present obvious differences in their vegetative or reproductive morphologies. This makes it necessary to use genetic approaches that account for the variability of the species.

The cactus family, with 1851 accepted species [16], is a diverse group within the Neotropics that presents remarkable diversity in life forms and reproductive strategies [17]. Additionally, this family is characterized by high levels of morphological convergence, where the most common life forms, such as columnar and globose forms, have evolved repeatedly across the Cactaceae. This phenomenon has largely complicated the taxonomy of the family, among which South American globose cacti are among the least studied [18]. The tribe Notocacteae (Cactoideae subfamily) harbors several globose species and is regarded as one of the oldest and most narrowly distributed lineages in southern South America [19]. Within this tribe, *Eriosyce sensu lato* has a complex taxonomic history with a high level of uncertainty, evidenced by the long history of taxonomic changes since its description more than 100 years ago. Species complexes, which include several populations, were historically assigned species ranks, but are currently considered single species. *Eriosyce curvispina*, with dozens of published names, is one of the most taxonomically complex species within the family [16]. This taxonomic uncertainty is due to the presence of taxa that, based on their morphology, cannot be discriminated, even when having long evolutionary histories that separate them. Additionally, several groups lack comprehensive samples of populations. This has a negative impact on our understanding of the origin and persistence mechanisms of the groups, and underestimates taxonomic diversity, challenging the efficiency of conservation and management actions.

The existence of complexes of barely distinguishable species is a well-known phenomenon in several groups of angiosperms (e.g., [20,21]). However, in Cactaceae, this phenomenon is just beginning to be understood after major revisions in the family that have lumped much of its diversity [22]. Accurate species delimitation is of great importance in establishing precise hypotheses about the mode and tempo of the evolutionary origins of species. In *E. curvispina*, two contrasting hypotheses can be delineated, a single origin with posterior divergence of infraspecific taxa, or independent speciation events that would reveal a greater diversity at the species level. Here, we investigate the phylogenetic relationships and molecular diversity across *E. curvispina* populations in order to delimit species and to understand the sequence of origin of its diversity. To achieve these objectives, we analyzed the phylogenetic relationships within *Eriosyce* section *Horridocactus*, the clade where *E. curvispina* is nested [19], and developed 12 microsatellites markers to further investigate the genetic structure of the species complex.

## 2. Materials and Methods

### 2.1. Plant Material

**Figure 1:**
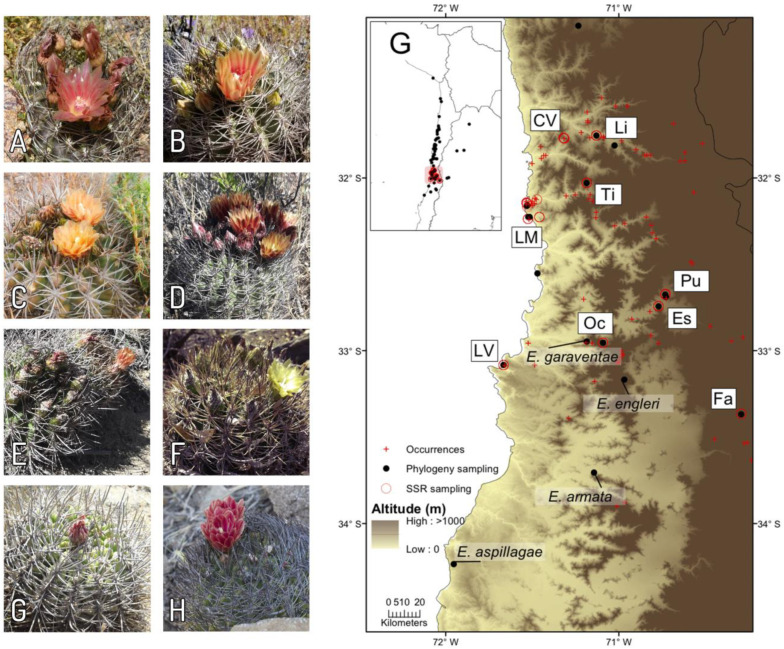
Morphological diversity of *Eriosyce curvispina* from nine populations sampled for phylogenetic and population evolutionary inferences.: (**A**), Choapa Valley (CV); (**B**), Tilama (Ti); (**C**), Los Molles (LM); (**D**), Putaendo (Pu); (**E**), Ocoa (Oc); (**F**), Escorial (Es); (**G**), Laguna Verde (LV); (**H**), Farellones (Fa). Photos: A by M. Rosas; B and C by P.C. Guerrero; D and E by H.M.Villalobos-Barrantes; F and G by B. M. Meriño and H by J. Keymer. The map in G shows locations of *E. curvispina* populations in central Chile: red crosses are occurrences; the black dots are the sampling sites used in phylogenetic analysis; the empty circles are the sampling sites used in population analysis.

The *E. curvispina* complex has a globular stem form, with slightly curved prickles; the forms of the flowers are also very similar, but the colors differ from place to place, from lemon yellow to reddish, as shown in Figure 1. They are produced from young areoles, forming a circle around the stem apex; flowers are broad, slightly funnelform, and of 3–5 cm (cm) long by 3–5 cm wide [23].

To analyze the evolutionary relationships of the *E. curvispina* complex, we added 33 samples to a backbone phylogenetic data matrix of the genus published elsewhere [19]. Specifically, we sequenced 18 new individuals of the section *Horridocactus* and 15 new individuals of the sister clade *Neoporteria*, all from field collected specimens. We mainly collected the material from roots or flowers and kept it in CTAB-NaCl (hexadecyltrimethylammonioum bromide-sodium chloride) buffer (2%:22%) to transport to the laboratory and store at 4 °C until the extraction.

For the microsatellite analysis, we sampled 150 individuals of the complex *Eriosyce curvispina* from nine populations: Putaendo, El Escorial, Tilama, Choapa Valley, Limahuida and Laguna Verde (15 individuals per site), Farellones (16 individuals), Ocoa (nine individuals) and Los Molles-Pichidangui (35 individuals), all collected as described in Table 1 and Figure 1.

### 2.2. Sequence-Based Phylogenetic Inferences

For DNA extraction, we used 40–50 mg of root or flower tissue that first was pulverized into a fine power using an automatic homogenizer, and then total DNA was extracted using a DNeasy Plant Kit (Qiagen, Valencia, CA, USA). For the phylogenetic analysis, we amplified three noncoding chloroplast markers (*rpl32-trnL*, *trnH-psbA* and *trnL-trnF*), one plastid gene (*ycf1*) and one nuclear gene (PHYC) following the protocol described by Guerrero et al. [19]. PCR products were checked on 1% agarose gels and then sent to Macrogen (Seoul, Korea) for sequencing in both directions.

We utilized 117 sequences (33 new and 84 sequences from Guerrero et al. [19]), assembled and edited in the program Geneious Prime^®^ 2020.2.3 (Biomatters Ltd., Auckland, New Zeland). Sequences for each marker were automatically aligned using Muscle and then checked manually. The outgroup consisted of 19 species, mostly from the core Notocacteae. Each marker was aligned separately and then concatenated. A microsatellite region in the *ycf1* dataset was excluded (450 bp) due to ambiguous alignment in this region. The best partitions and molecular models were evaluated using PartitionFinder v.2.1.1, as described by Guerrero et al. [19].

Bayesian inference of the concatenated matrix was performed using Mr.Bayes v3.2.7 [24], and unlinked rate heterogeneity, based on frequencies and substitution rates across partitions. Bayesian ran 30 million generations across four independent runs with four chains each, sampling every 1000 generations. The best models were GTRG for *rpl32-trnL* and *trnH-psbA* and GTRINVGAMMA for the rest of the markers. Convergence was monitored using the standard deviation of split frequencies, and when this value stabilized below 0.01, it was considered a strong indication of convergence. The associated likelihood values, effective sample size (ESS) values, and burn-in values of the different runs were verified with the program Tracer v1.7.1 [25]. Trees were visualized using software FigTree v1.4.4 [26] (Supplementary Figure S1).

**Figure 2:**
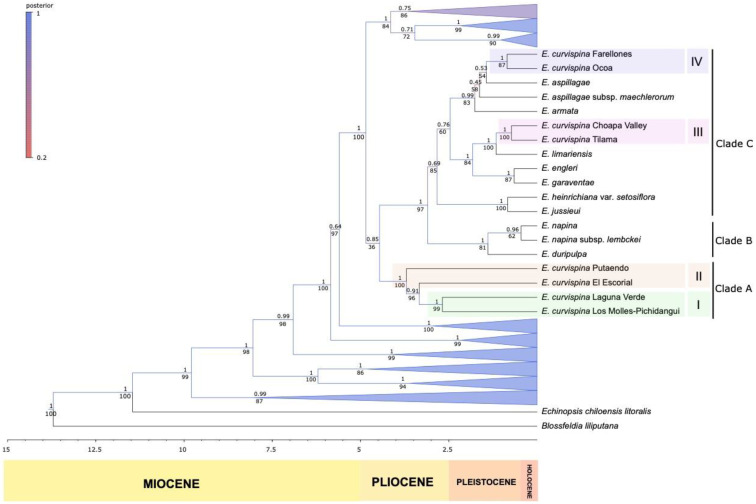
Time-calibrated phylogeny of *Eriosyce* with internal clades collapsed, except for the *Horridocactus* clade, to facilitate visualization of the phylogenetic position of the putative members of *E. curvispina*. Bayesian posterior probabilities are shown above branches, and maximum-likelihood bootstrap support values are shown below branches. Groups I to IV are based on the sequence-based phylogenetic inferences with five molecular markers (Section 2.2).

Divergence dates were estimated using BEAST v2.6.2 [27], and were evaluated with the four clock models available on a concatenate matrix considering one sample per species (87 taxa). The best clock model was the relax clock exponential determined with the value of marginal L provided by PathSampler (an application for model selection inside the BEAST package). For the prior, we used the nodes’ age information reported in the Cactaceae phylogeny by Hernández-Hernández et al. [17], with 95% highest posterior density (HPD). The three dated nodes we used in our analysis were: (i) the root node corresponding to 17.15 Ma with normal distribution between 12.67 and 24.46 Ma, (ii) the second node corresponding to 12.44 Ma with normal distribution between 8.59 and 17.95 Ma and (iii) the third node (Core Notocacteae) corresponding to 8.78 with normal distribution between 5.54 and 13.03. Trees were visualized using the software FigTree v1.4.4 (see Figure 2 and Supplementary Figure S2). Maximum likelihood (ML) analyses of this concatenated matrix were performed using the program raxmlGUI 2.0 v.2.0.6 [28]. The search for an optimal ML tree run was combined with a rapid bootstrap analysis based on 100 trees and 1000 replicates (see Figure 2 and Supplementary Figure S3).

### 2.3. Microsatellite-Based Population Genetics

Microsatellites were designed on the basis of next-generation sequencing from four DNA pools (Pool A *Eriosyce chilensis* var. *albidiflora*; Pool C *E. chilensis*; Pool M *E. curvispina* and Pool S *E. litoralis*). These samples were collected between Los Molles and Pichidangui localities (Latitude −32.2 and Longitude −71.47). Sequencing libraries were prepared using a Nextera XT DNA kit, and then sequenced using an Illumina^®^ MiSeq Next Generation Sequencer with an output of 15 million fragment reads at AutralOmics facilities (https://australomics.cl/, accessed on 25 January 2022 [29]). After sequencing, the first step was removing adapters, checking read quality and cleaning read quality using Trimmomatic [30] and Prinseq [31] software, with a Q > 28. The second step was assembling paired reads with Pandaseq software [32], in order to obtain longer fragments. The third step was the identification of repetitive sequences and the generation of different sets of partitions for each identification; for this purpose, the virtual machine of the QDD software [33] was used, which by way of Primer3 [34] generated a set of partitions for each repetitive sequence identified. Finally, a BLAST (Basic Local Alignment Search Tool [35]) search of the amplicons generated for each microsatellite identified for each sample analyzed was performed, to find the common sequences in all pools.

From the information described above, we choose dinucleotide and trinucleotide sequences common in the four pools. Afterwards, a set of 52 primer pairs were tested with different *Eriosyce* DNA samples to find the most polymorphic ones. Finally, we choose 12 loci: PS5, PS9, PC2, PC11, PM10, PA12, PM8, PC10, PM6, PC7, PM7 and PA6 (Supplementary Table S1) to perform three multiplex with four primers each. PCR amplifications were set up in 13 uL volume composed of 1ul of DNA samples diluted at 0.5 ng/ul, 6.6 ul of 2X of Master Mix SapphireAmp Fast PCR^®^, 4,4 ul of Water (Takara Bio, San Jose, CA, USA, Inc.) and 0.5 uL of each primer at 5uM. The reverse primers were 5′fluorescently labeled with either 6-Carboxyfluorescein(6-FAM), 2′-chloro-7′phenyl-1,4-dichloro-6-carboxy-fluorescein (VIC), 2′-chloro-5′-fluoro-7′,8′-benzo-1,4-dichloro-6-carboxyfluorescein (NED) or PET (unplublished propietary of Applied Biosystems, San Francisco, CA, USA).

The PCR’s were performed in a thermo-cycler (Veriti 96 Well Thermal Cycler, Applied Biosystems) programmed as: 1 min at 94 °C for initial denaturation, followed by 35 cycles of 98 °C for 00.05 s, primer specific annealing temperature (58 or 62 °C) for 00.05 s min, 72 °C for 00:40 s, and final extension at 72 °C for 1:45 min. The PCR products were checked on 1% agarose gels in 1X Tris-boric acid-EDTA buffer running a mixture of 5 ul of PCR product with 2 ul of 6X loading buffer (New England Biolabs, Hitchin, UK) with GelRed^®^ (Biotium, USA) and co-running with a 100 bp DNA ladder (New England Biolabs, UK). The amplified PCR products were sent to the Unidad de Secuenciación y Tecnologías Ómicas at the Pontificia Universidad Católica de Chile (Santiago, Chile) for genotyping. They were analyzed on ABI PRISM 3500 XL (Applied Biosystems) using the size standard Genescan LIZ 500 (Applied Biosystems, San Francisco, CA, USA). Then the loci from dinucleotides repeats, were scored manually and analyzed using Geneious Prime^®^ (Geneious software v2020.2.3, Biomatters Ltd., Auckland, New Zeland) with Microsatellite plugin taking only the fragments over 100 pb.

We estimated allelic frequencies, polymorphic loci (%P), expected heterozygosity (He) and observed heterozygosity (Ho) with GenAlEx 6.51b2 [36]; the fixation index (*Fis*) and linkage disequilibrium (LD) with Genetix 4.05; *Fst* with FreeNA [37]; and the genetic variation within populations, as well as among populations and regions (phylogenetic I-IV Groups), with Analysis of Molecular Variance (AMOVA) using GenoDive v.3.0 [38] (Table 2).

**Figure 3:**
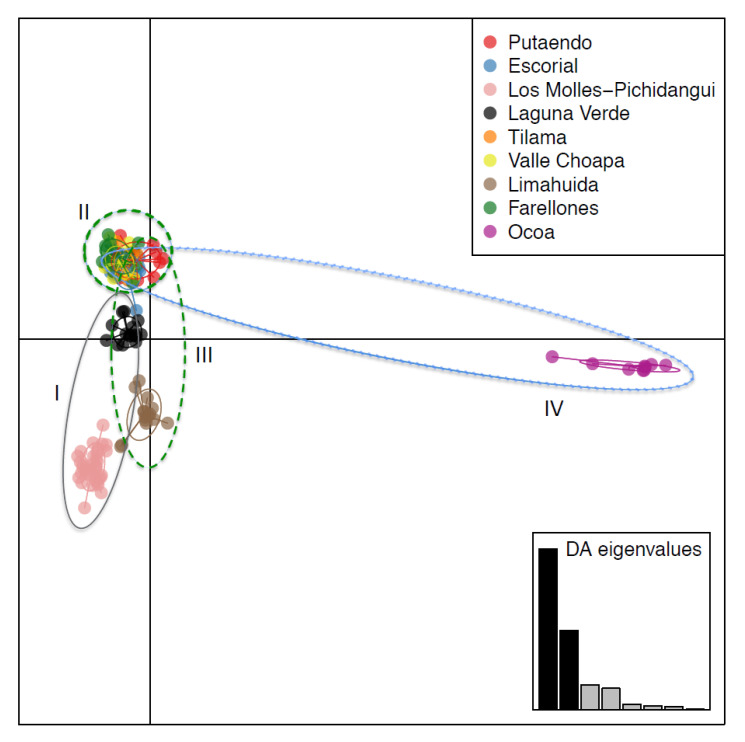
DAPC analysis for 150 individuals of *Eriosyce curvispina* based on 12 microsatellites loci. Ellipsoids depict Groups I–IV identified in the Bayesian phylogenetic inference in Figure 2.

The population’s genetic structure was evaluated using Structure v2.3.4 [39]; this approach consists of plotting the second-order rate change in ln Pr (X/K) for successive K_s_ (referred as DK) against a range of K values and selecting the true K based on the maximal value using the Evanno method [40] (see Supplementary Figure S4). We used a set of K from 2 to 10, with 1 million runs and 10 runs per K and the package pophelper in R to analyze and visualize the population structure results [41,42]. In addition, we used the Discriminant Analysis of Principal Components (DAPC) to evaluate the genetic structure in the library Adegent [43] (see Figure 3). We used cross-validation to determine the appropriate number of components to retain; in our case, we retained 50 principal components, accounting for 0.793 of the proportion of the variance conserved.

## 3. Results

### 3.1. Phylogenetic Reconstruction

The concatenated matrix with the five markers included 4841 bp of aligned sequences for 117 individuals, of which 2440 were variable for the complete matrix and 1958 for the ingroup (Table 3). Of the aligned concatenated matrix, the plastid non-coding marker *rpl32-trnL* contributed 1354 bp (30 % of variable sites), *trnL-trnF* contributed 1084 bp (15%) and *trnH-psbA* contributed 439 bp (4%)**,** while the plastid gene *ycf1* contributed 930 bp (20%) and the nuclear gene PHYC contributed 1034 bp (locus with 31% of variable sites).

Sequence data yielded a well-supported phylogenetic hypothesis of the Horridocactus clade (Figure 2; Supplementary Figures S1–S3). Bayesian and ML inferences retrieved identical topologies of the Horridocactus clade (Supplementary Figures S2 and S3), and strongly support the non-monophyly of the *E. curvispina* complex; we found that its members clustered in four pairs of taxa distributed across three different clades within Horridocactus in four groups (I-IV, Figure 2; Supplementary Figures S1–S3). These groups branched along the phylogenetic tree in a sequence with independent timing of origin (Figure 2; Supplementary Figure S2). The oldest divergence occurred in Clade A at 3.7 ± 2.3–5.5 Ma (95% confidence interval, IC), separating *E. curvispina* (Los Molles and Laguna Verde) from Andean populations Putaendo and Escorial. Within Groups I and II, divergences were dated 3.3 ± 2.0–4.9 Ma and dated 2.7 + 1,6–3.8 Ma, respectively. Non-putative members of the *E. curvispina* complex were retrieved in Clade B, which is composed of species with the geophyte growth form (northernmost distribution within Horridocactus). Within Clade C, Group III of *E. curvispina* harbors populations from Choapa Valley, Tilama and Limahuida, which were placed together in a branch sister to *E. limariensis*; this divergence occurred at dated 1.2 + 0.5–1.9 Ma. Two more members of the *E. curvispina* complex (Ocoa and Farellones) originated at 1.4 ± 0.4–2.1 Ma (Group IV), and were placed sister to *E. aspillagae*, which was the species with southernmost distribution.

### 3.2. Genetic Diversity and Differentiation of Species

The 12 nuclear simple sequence repeats (SSRs) markes revealed that the groups previously identified in the phylogenetic inferences differ in their genetic diversity and structure (Table 2). We detected an overall high percent of polymorphic loci, expected heterozygosity, effective alleles and Shannon’s information index. Within Groups I–IV, the differentiation values (*Fst* = Wright´s F-statistics) were significantly different from zero. The population in Ocoa has little genetic variation, shown by the lowest fixation index, observed and expected heterozygosity, and levels of polymorphism of 67%**,** also present a fixation index, *Fis* = 0.0990 (see Table 2).

The DAPC analyses showed a range of variation among populations, with less divergence between Group II (*E. curvispina* Putaendo and *E. curvispina* Escorial) and Group III (*E. curvispina* from Choapa Valley, Tilama and Limahuida). Group I presented substantial genetic differentiation compared to its closely related Groups II and III. Group IV (*E. curvispina* Ocoa and Farellones) showed a higher genetic divergence, as the population of *E. curvispina* at Ocoa is the most differentiated genetic group (Figure 3). Structure analysis also supported these results (Supplementary Figure S3).

AMOVA analysis indicated that the majority of genetic variation occurred within populations (43.4%; Table 1), less among populations and even less among regions, with a non-significant coefficient of correlation value (*p*-value = 0.208). These results are consistent with the DAPC analysis, where the cluster formed by *E. curvispina* from Putaendo, El Escorial, Farellones, Tilama and Valle del Choapa was observed, and with the result of Structure, K = 6 (Supplementary Figure S3).

## 4. Discussion

Phylogenetic inferences retrieved a well-supported phylogenetic tree revealing evolutionary lineages within the section *Horridocactus* and strong divergences among putative members of the *E. curvispina* complex. There is support for independent speciation events, revealing a greater diversity that is going unnoticed, although important genetic uniqueness has been cumulated. Horridocactus species were grouped into three major clades (A–C); two of them harbor *E. curvispina* populations, which in turn were distributed into four distinct groups (I–IV). This revealed a more complicated evolutionary scenario that is not consistent with the existence of a taxonomic complex, because it presents independent cladogenetic events. Additionally, our study strongly suggests that the members of Groups I to IV originated at different time spans, supporting that divergence mechanisms produced deep and shallow speciation events across the evolutionary time.

The four groups identified are geographically isolated either by latitudinal or altitudinal separation; the high topographic complexity in the central zone of Chile means that, even in short linear distances between populations, they can have strong reproductive isolation [44]. Within Groups I and II, in Clade A (Figure 2), we found significant genetic cumulative divergence, as can be inferred by the great branch length observed in the phylogram between the species. The severe climatic changes that occurred at the beginning of the Pleistocene, together with the complex topography in central Chile, might have had a strong impact on the early divergence of these taxa. For instance, the establishment of new climate regimes and/or permanent ice barriers would have played a key role in this separation [45,46,47], where Andean populations were able to remain in refugia, fostering spatial reproductive isolation. This phenomenon has been suggested in other Andean plant clades with important divergences at the beginning of the Pleistocene [48,49,50]. The coastal populations of *E. curvispina* at Los Molles and Laguna Verde presented an estimated divergence of 3.3 Ma; additionally, DAPC of SSRs data support the existence of the population genetic structure, suggesting that there is a hard barrier between these two coastal populations that constrain gene flow, reinforcing differences. In contrast, the Andean populations (Escorial and Putaendo) of Clade A showed high levels of divergence in the phylogenetic tree (Supplementary Figure S2), while the population genetic structure assessed by microsatellites data was less significant. These distinct patterns of genetic variation obtained from different molecular markers suggest that modern genetic exchange inferred from microsatellites data may have reduced the genetic distance between these two taxa [51,52,53,54].

Members of Groups III and IV originated more recently (0.85–0.72 Ma) in two independent cladogenetic events. Group III occupies valleys of north central Chile, while species members of Group IV occupy areas further south within the transversal mountain ranges and the Andes. Both habitat types have experienced major vegetation changes over the last million years following glacial cycles [55,56,57,58]; these long-term cycles, with the effect of the South American dry diagonal, have significant xerophytic effects, favoring range expansion and contractions in plant populations [59,60,61,62]. Mechanisms contributing to isolation and posterior divergence involve the complex topography of central Chile, and the bee pollination system, which can easily lead to reproductive isolation, as most native bees are small in size and have a reduced pollination range [63]. In addition, this divergence between coastal and inland populations might be attributed to asymmetric selection regimes exerted by different climate regimes at different elevations [50,51,52] and to the distinct pollinator guilds, which covary with elevation [64].

Members of section *Neoporteria*, another group of globose cacti, diverged along the coast but did not show levels of deep divergence [19]; this difference is probably due to differences in pollination systems. While species of the Horridocactus clade are bee-polinated [65], most of the Neoporteria species are hummingbird pollinated [66,67]. The high vagility of hummingbirds would allow pollen exchange over greater distances, reducing genetic divergence.

The population at Ocoa showed the lowest fixation index, and the lowest genetic diversity among populations, congruent with the fact that it is an isolated population, and thus gene flow with other populations is less likely. This isolation may be caused by the fact that the native xerophitic vegetation remains in island-like hills, and in the last two centuries, the surrounded matrix has been transformed into an anthropized landscape due to different activities such as cereal crops, fruits, livestock, and housing [68,69]. In consequence, local “*E. curvispina*” has experienced a habitat reduction, increasing the chances for inbreeding, plant extirpation and genetic diversity reduction.

These results support inferences about the historical and modern eco-evolutionary processes that have contributed to the observed diversity patterns of this group, as the combination of geographic factors (topography and climatic variations) added to pollination guild variation, and in more recent times agricultural activities, may be major factors molding the genetic diversity of these globular cacti. Despite the wide distribution and importance of the arid and semi-arid zones, phylogenetic and population genetics studies are still scarce [70,71,72], especially those that address the mechanisms by which they enabled the genetic diversity that we see today [73,74,75,76,77].

## 5. Conclusions

The phylogenetic reconstruction with chloroplast DNA (cpDNA) and nuclear DNA markers showed strong relationships of the taxa within *Eriosyce*, and that the *Eriosyce curvispina* complex is a polyphyletic group lumping at least four evolutionary lineages. We identified four groups that originated in independent cladogenetic events occurring at different temporal depths; these groups presented high genetic diversity, and their populations were genetically structured. These results improve our understanding on the origin of an endemic rich group of cacti, allow us to more clearly determine its biodiversity, and suggest that a fraction of the flora of the Mediterranean Central Chile biodiversity hotspot may be cryptic. This information is mandatory for accurate extinction risk assessments and the efficient design of conservation actions, avoiding overlooking highly threatened species in an increasingly anthropized landscape.

## Figures and Tables

**Table 1:** Analysis of molecular variance (AMOVA) based on 12 microsatellites. Abbreviations are as follows: %var = percent of variance; Std.Dev. = standard deviation; *p*-value = probability.

Source of Variation	Nested in	%Var	Std.Dev.	*p*-Value
Within Individuals	-	43.4	0.042	-
Among Individuals	Population	38.2	0.042	0
Among Populations	Region	17.9	0.023	0
Among Regions	-	0.5	0.008	0.208

**Table 2:** Genetic estimators for populations of the *Eriosyce curvispina* complex. Abbreviations are as follows: N = number of samples; Na = allele number; Ne = number of effective alleles; I = Shannon’s information index; Ho = observed heterozygosity; He = expected heterozygosity; uHe = unbiased expected heterozygosity; *Fis* = fixation index; %P = percentage of polymorphic loci; HW = Hardy–Weinberg equilibrium (multilocus per population).

Location	Latitude	Longitude	N	Na	Ne	I	Ho	He	uHe	*Fis*	%P	HW
Putaendo	−32.67	−70.73	15	7.167	4.481	1.547	0.467	0.689	0.713	0.3537	100%	0.354
El Escorial	−32.74	−70.77	15	8.250	5.340	1.819	0.391	0.795	0.823	0.5342	100%	0.534
Los Molles Pichidangui	−32.17	−71.47	34	11.583	6.003	1.994	0.361	0.807	0.819	0.5634	100%	0.563
Laguna Verde	−33.08	−71.67	13	7.333	4.798	1.682	0.375	0.760	0.790	0.5359	100%	0.537
Tilama	−32.03	−71.18	15	7.750	5.370	1.640	0.425	0.707	0.732	0.4291	92%	0.429
Choapa Valley	−31.77	−71.32	15	7.083	4.070	1.435	0.400	0.645	0.668	0.4093	100%	0.409
Limahuida	−31.75	−71.13	15	10.750	6.235	2.040	0.570	0.822	0.851	0.3381	100%	0.338
Farellones	−33.37	−70.29	15	6.667	4.562	1.529	0.332	0.697	0.723	0.5502	100%	0.55
Ocoa	−32.95	−71.09	9	1.667	1.450	0.367	0.241	0.250	0.265	0.0990	67%	0.099

**Table 3:** Statistics for the 117-sample DNA sequence alignments.

Locus	Total Length	Ingroup Variable Characters	Total Variable Characters	Parsimony Informative Characters	Ingroup Coverage (%)	Outgroup Coverage (%)
*rpl32-trnL*	1354	622	743	347	16	83
*trnL-trnF*	1084	226	363	206	16	83
*trnH-psbA*	439	34	86	33	16	84
*ycf1*	930	343	499	303	16	83
PHYC	1034	733	749	271	70	87
Concatenated matrix	4841	1958	2440	1160		

## Data Availability

The datasets presented in this study can be found in online repositories. The names of the repository/repositories and accession number(s) can be found at the following link: https://github.com/pabloguerrero-cmd/Villalobos-Barrantes_etal_Genes_2022, accessed on 20 January 2022.

## Supplementary Materials

The following supporting information can be downloaded at https://www.mdpi.com/article/10.3390/genes13020240/s1, Figure S1: Phylogeny of *Eriosyce s.l.* inferred with Mr.Bayes with 30 million generations, each node with *posteriori* probabilities; Figure S2: Phylogeny of *Eriosyce s.l.* with 87 taxa obtained using Beast2 with Relax Clock Exponential Model, each node with height 95% HPD; Figure S3: Phylogeny of *Eriosyce s.l.* with 87 taxa obtained using RaxML with 1000 repeats, each node with maximum-likelihood bootstrap support; Figure S4: Evanno plot for 150 taxa of *Eriosyce curvispina* complex with 12 microsatellites loci; Table S1: Primers for amplification and multiplexing of microsatellites.

## References

[1] Rice J., Seixas C.S., Zaccagnini M.E., Bedoya-Gaitán M., Valderrama N., IPBES (2018). The IPBES Regional Assessment Report on Biodiversity and Ecosystem Services for the Americas.

[2] Goettsch B., Hilton-Taylor C., Cruz-Piñón G., Duffy J.P., Frances A., Hernández H.M., Inger R., Pollock C., Schipper J., Superina M. (2015). High proportion of cactus species threatened with extinction. Nat. Plants.

[3] Arroyo M.T.K., Marquet P., Marticorena C., Somoneti J., Cavieres L., Sequeo F., Rozzi R., Massardo F., Ugalde R.J., Stutzin M. (2008). El hostpot chileno, prioridad mundial para la conservación. Biodiversidad de Chile, Patrimonio y Desafíos.

[4] Cowling R.M., Rundel P.W., Lamont B.B., Arroyo M.K., Arianoutsou M. (1996). Plant diversity in mediterranean-climate region. Trends Ecol. Evol..

[5] Myers N., Mittermeier R., Mittermeier C., Fonseca G., Kent J. (2000). Biodiversity hotspots for conservation priorities. Nature.

[6] Urbina M.A., Guerrero P.C., Jerez V., Lisón F., Luna-Jorquera G., Matus-Olivares C., Ortiz J.L., Pavez G., Pérez-Alvárez M.J., Riquelme-Bugueño R. (2021). Extractivist polices hurt Chile’s ecosystems. Science.

[7] Finot V., Baeza C., Muñoz-Schick M., Ruiz E., Espejo J., Alarcón D., Carrasco P., Novoa P., Eyzaguirre M.T. (2018). Guía de Campo Alstroemerias Chilenas.

[8] Rojas G., Baeza C. (2021). *Alstroemeria esteparica* (Alstroemeriaceae) una nueva especie para la flora del Cono Sur de Sudamérica. Gayana Bot..

[9] Cádiz-Véliz A. (2021). *Miersia putaendensis* sp. nov. (Gilliesieae, Amaryllidaceae), a new species endemic to Central Chile. Phytotaxa.

[10] García N., Meerow A.W., Arroyo-Leuenberger S., Oliveira R.S., Dutilh J.H., Soltis P.S., Judd W.S. (2019). Generic classification of Amaryllidaceae tribe Hippeastreae. Taxon.

[11] Toro-Núñez O., Al-Shehbaz I.A., Mort M.R. (2015). Phylogenetic study with nuclear and chloroplast data and ecological niche reveals *Atacama* (Brassicaceae), a new monotypic genus endemic from the Andes of the Atacama Desert, Chile. Plant Syst. Evol..

[12] Bravo-Monasterio P., Baeza-Horta G., Peñailillo P., Alarcón D., Contreras D. (2014). Una nueva especie del género *Bipinnula* (Orchidaceae) para Chile. Gayana Bot..

[13] Gurvish D.E., Zeballos S.R., Demaio P.H. (2014). Diversity and composition of cactus species along and altitudinal gradient in the Sierras del Norte Mountains (Córdoba, Argentina). S. Afr. J. Bot..

[14] Ortega-Baes P., Sühring S., Sajama J., Sotola E., Alonso-Pedano M., Bravo S., Godínez-Alvarez H., Ramawat K. (2010). Diversity and Conservation in the Cactus Family. Desert Plants.

[15] Bickford D., Lohman D.J., Sodhi N.S., Ng P.K., Meier R., Winker K., Ingram K.K., Das I. (2007). Cryptic species as a window on diversity and conservation. Trends Ecol. Evol..

[16] Korotkova N., Aquino D., Arias S., Eggli U., Franck A., Gómez-Hinostrosa C., Guerrero P.C., Hernández H.M., Kohlbecker A., Köhler M. (2020). Cactaceae at Caryophyllales.org—A dynamic online species-level taxonomic backbone for the family. Willdenowia.

[17] Hernández-Hernández T., Brown J.W., Schlumpberger B.O., Eguiarte L.E., Magallón S. (2014). Beyond aridification: Multiple explanations for the elevated diversification of cacti in the New World Succulent Biome. New Phytol..

[18] Guerrero P.C., Majure L.C., Cornejo-Romero A., Hernández- Hernández T. (2019). Phylogenetic Relationships and Evolutionary Trends in the Cactus Family. J. Hered..

[19] Guerrero P.C., Walter H.E., Arroyo M.T.K., Peña C.M., Tamburrino I., De Benidictis M., Larridon I. (2019). Molecular phylogeny of the large South American genus *Eriosyce* (Notocacteae, Cactaceae): Generic delimitation and proposed changes in infrageneric and species ranks. Taxon.

[20] Henriquez C.L., Arias T., Pires J.C., Croat T.B., Schaal B.A. (2014). Phylogenomics of the plant family Araceae. Mol. Phylogenet. Evol..

[21] Grant V. (1981). Plant Speciation.

[22] Hunt D.R., Taylor N., Charles G. (2006). The New Cactus Lexicon.

[23] Katermann F. (1994). Eriosyce (Cactaceae) the Genus Revised and Amplifed.

[24] Ronquist F., Teslenko M., Van der Mark P., Ayres D.L., Darling A., Höhna S., Larget B., Liu L., Suchard M.A., Huelsenbeck J.P. (2012). MrBayes 3.2: Efficient Bayesian phylogenetic inference and model choice across a large model space. Syst. Biol..

[25] Rambaut A., Drummond A.J., Xie D., Baele G., Suchard M.A. (2018). Posterior summarization in Bayesian phylogenetics using Tracer 1.7. Syst. Biol..

[26] Rambaut A. (2016). FigTree v1.4.4. Institute of Evolutionary Biology, University of Edinburgh, Edinburgh. http://tree.bio.ed.ac.uk/software/figtree/.

[27] Bouckaert R., Heled J., Kühnert D., Vaughan T., Wu C.-H., Xie D., Suchard M.A., Rambaut A., Drummond A.J. (2014). BEAST 2: A Software Platform for Bayesian Evolutionary Analysis. PLoS Comput. Biol..

[28] Edler D., Klein J., Antonelli A., Silvestro D. (2021). raxmlGUI 2.0: A graphical interface and toolkit for phylogenetic analyses using RAxML. Methods Ecol. Evol..

[29] AUSTRAL-Omics Core Facility of Universidad Austral de Chile. https://australomics.cl/.

[30] Bolger A.M., Lohse M., Usadel B. (2014). Trimmomatic: A flexible trimmer for Illumina Sequence Data. Bioinformatics.

[31] Schmieder R., Edwards R. (2011). Quality control and preprocessing of metagenomic datasets. Bioinformatics.

[32] Masella A.P., Bartram A.K., Truszkowski J.M., Brown D.G., Neufeld J.D. (2012). PANDAseq: Paired-end assembler for illumina sequences. BMC Bioinform..

[33] Meglécz E., Pech N., Gilles A., Dubut V., Hingamp P., Trilles A., Grenier R., Martin J.F. (2014). QDD version 3.1: A user friendly computer program for microsatellite selection and primer design revisited: Experimental validation of variables determining genotyping success rate. Mol. Ecol. Resour..

[34] Rozen S., Skaletsky H., Krawetz S., Misener S. (2000). Primer3 on the WWW for general users and for biologist programmers. Bioinformatics Methods and Protocols: Methods in Molecular Biology.

[35] Altschul S.F., Gish W., Miller W., Myers E.W., Lipman D.J. (1990). Basic local alignment search tool. J. Mol. Biol..

[36] Peakall R., Smouse P.E. (2012). GenAlEx 6.5: Genetic analysis in Excel. Population genetic software for teaching and research-an update. Bioinformatics.

[37] Chapuis M.P., Estoup A. (2007). Microsatellite null alleles and estimation of population differentiation. Mol. Biol. Evol..

[38] Meirmans P.G. (2020). GENODIVE version 3.0: Easy-to-use software for the analysis of genetic data of diploids and polyploids. Mol. Ecol. Resour..

[39] Pritchard J.K., Stephens M., Donnelly P. (2000). Inference of population structure using multilocus genotype data. Genetics.

[40] Evanno G., Regnaut S., Goudet J. (2005). Detecting the number of clusters of individuals using the software structure: A simulation study. Mol. Ecol..

[41] Francis R.M. (2017). pophelper: An R package and web app to analyse and visualize population structure. Mol. Ecol. Resour..

[42] R Core Team (2021). R: A Language and Environmental for Statistical Computing.

[43] Jombart T. (2008). Adegenet: A R package for the multivariate analysis of genetic markers. Bioinformatics.

[44] Rozzi R., Arroyo M.K., Armesto J.J. (1997). Ecological factors affecting gene flow between populations of *Anarthrophyllum cumingii* (Papilionaceae) growing on equatorial- and polar-facing slopes in the Andes of Central Chile. Plant Ecol..

[45] Böhnet T., Weigend M., Merklinger F., Quandt D., Luebert F. (2020). Historical assembly of Zygophyllaaceae in Atacama Desert. Front. Biogeogr..

[46] Merklinger F., Böhnert T., Arakaki M., Weigend M., Quandt D., Luebert F. (2021). Quaternary diversification of a columnar cactus in the driest place on earth. Am. J. Bot..

[47] Bonatelli I.A.S., Perez M.F., Peterson A.T., Taylor N.P., Zappi D.C., Machado M.C., Koch I., Pires A.H., Moraes E.M. (2014). *Pilosocereus aurisetus* and allies. Mol. Ecol..

[48] Glade-Vargas N.S., Rojas C., Jara-Arancio P., Vidal P., Arroyo M.T.K., Hinojosa L.F. (2021). Biogeography of *Argylia* D. Don (Bignoniaceae): Diversification, Andean uplift and niche conservatism. Front. Plant Sci..

[49] Amaral D.T., Minhós-Yano I., Oliveira J.V.M., Romeiro-Brito M., Bonatelli I.A.S., Taylor N.P., Zappi D.C., Moraes E.M., Eaton D., Franco F.F. (2021). Tracking the xeric biomes of South America: The spatiotemporal diversification of Mandacaru cactus. J. Biogeogr..

[50] Ornelas J.F., Rodríguez-Gómez F. (2015). Influence of Pleistocene glacial/interglacial cycles on the genetic structure of the Mistletoe Cactus *Rhipsalis baccifera* (Cactaceae) in Mesoamerica. J. Hered..

[51] Rose L.A., Rieseberg L.H. (2013). Divergence is focused on few genomic regions early in speciation: Incipient speciation of sunflowers ecotypes. Evolution.

[52] Zalapa J.E., Price D.L., Kaeppler S.M., Tobias C.M., Okada M., Casler M.D. (2011). Hierarchical classification of switchgrass genotypes using SSR and chloroplast sequences: Ecotypes, ploidies, gene pools, and cultivars. Theor. Appl. Genet..

[53] Suo Z., Zhang C., Zheng Y., Zheng Y., He L., Jin X., Hou B., Li J. (2012). Revealing genetic diversity of tree peonies at micro-evolution level with hyper-variable chloroplast markers and floral traits. Plant Cell Rep..

[54] Li W., Liu Y., Yang Y., Xie X., Lu Y., Yang Z., Jin X., Dong W., Suo Z. (2018). Interspecific chloroplast genome sequence diversity and genomic resources in *Diospyros*. BMC Plant Biol..

[55] Li B., Lin F., Huang P., Guo W., Zheng Y. (2020). Development of nuclear SSR and chloroplast genome markers in diverse *Liriodendron chinense* germplasm based on low-coverage whole genome sequencing. Biol. Res..

[56] Hinojosa F., Villagrán C. (1997). Historia de los bosques del sur de Sudamérica, I: Antecedentes paleobotánicos, geológicos y climáticos del Terciario del cono sur de América. Rev. Chil. Hist. Nat..

[57] Villagrán C., Hinojosa F. (1997). Historia de los bosques del sur de Sudamérica, II: Análisis fitogeográfico. Rev. Chil. Hist. Nat..

[58] Hinojosa F., Villagrán C., Armesto J.J. (2006). Are Chilean coastal forests pre-Pleistocene relicts? Evidence from foliar physiognomy, palaeoclimate, and phytogeography. J. Biogeogr..

[59] Hinojosa F., Pérez F., Rougier D., Villagrán C., Armesto J.J., Montecinos V., Orlando J. (2015). Legados históricos de la vegetación de bosque en Chile. Ciencias Ecológicas 1983–2013: Treinta Años de Investigaciones Chilenas.

[60] Ossa C.G., Montenegro P., Larridon I., Pérez F. (2019). Response of xerophytic plants to glacial cycles in southern South America. Ann. Bot..

[61] Luebert F. (2021). The two South American dry diagonals. Front. Biogeogr..

[62] Bacon C.D., Velásquez-Puentes F.J., Hinojosa L.F., Schwartz T., Oxelman B., Pfeil B., Arroyo M.T.K., Wanntorp L., Antonelli A. (2018). Evolutionary persistence in *Gunnera* and the contribution of southern plant groups to the tropical Andes biodiversity hotspot. PeerJ.

[63] Guerrero P.C., Durán A.P., Walter H.E. (2011). Latitudinal and altitudinal patterns of the endemic cacti from the Atacama desert to Mediterranean Chile. J. Environ..

[64] Arroyo M.T.K., Primack R., Armesto J. (1982). Community studies in pollination ecology in the high temperate Andes of central Chile. I. Pollination mechanism and altitudinal variation. Am. J. Bot..

[65] Guerrero P.C., Antinao C.A., Vergara-Meriño B., Villagra C.A., Carvallo G.O. (2019). Bees may drive the reproduction of four sympatric cacti in a vanishing coastal mediterranean-type ecosystem. PeerJ.

[66] Walter H. (2008). Floral biology, phylogeography and systematics of *Eriosyce* subgenus *Neoporteria* (Cactaceae). Bradleya.

[67] Cádiz-Véliz A., Verdessi F., Carballo G.O. (2021). Shrub canopy matrix decreases reproductive output of a sheltered plant via pollinator exclusion. Basic Appl. Ecol..

[68] Dominguéz J.I., Vergara M.M., Aguirre R., Barrera D., Montero J., Cáseres L., Egullor P., Espinoza A., García A., Reyes A. (2019). Panorama de la Agricultura Chilena. https://www.odepa.gob.cl/.

[69] Síntesis Agropecuaria-Encuestas Intercensales Agropecuarias 2020–2021. https://www.ine.cl/estadisticas/economia/agricultura-agroindustria-y-pesca.

[70] Guerrero P.C., Arroyo M.T.K., Bustamante R.O., Duarte M., Hagemann T.K., Walter H.E. (2011). Phylogenetics and predictive distribution modeling provide insights into the geographic divergence of *Eriosyce* subgen. *Neoporteria* (Cactaceae). Plant Syst. Evol..

[71] Koch M.A., Kleinpeter D., Auer E., Siegmund A., del Rio C., Osses P., García J.-L., Marzol M.V., Georg Zizka G., Kiefer C. (2019). Living at the dry limits: Ecological genetics of *Tillandsia landbeckii* lomas in the Chilean Atacama Desert. Plant Syst. Evol..

[72] Larridon I., Walter H.E., Guerrero P.C., Duarte M., Cisternas M.A., Peña-Hernández C., Bauters K., Asselman P., Goetghebeur P., Samarin M.S. (2015). An integrative approach to understanding the evolution and diversity of *Copiapoa* (Cactaceae), a threatened endemic Chilean genus from Atacama Desert. Am. J. Bot..

[73] Gutiérrez-Flores C., García-De León F.J., León-De la Luz J.L., Cota-Sánchez J.H. (2016). Microsatellite genetic diversity and mating systems in the columnar cactus *Pachycereus pringlei* (Cactaceae). Perspect. Plant Ecol. Evol. Syst..

[74] Cavender-Bares J. (2019). Diversification, adaptation, and community assembly of the American oaks (*Quercus*), a model clade for integrating ecology and evolution. New Phytol..

[75] Toro-Núñez O., Lira-Noriega A. (2020). Discordant phylogenetic endemism patterns in a recently diversified Brassicaceae lineage from the Atacama Desert: When choices in phylogenetics and species distribution information matter. J. Biogeogr..

[76] Silva G.A.R., Antonellli A., Lendel A., de Moraes E.M., Manfrin M.H. (2018). The impact of early Quaternary climate change on the diversification and population dynamics of a South American cactus species. J. Biogeogr..

[77] Arakaki M., Christin P.A., Nyffeler R., Lendel A., Eggli U., Ogburn R.M., Spriggs E., Moore M.J., Edwards E.J. (2011). Contemporaneous and recent radiations of the world’s major succulent plant lineages. Proc. Natl. Acad. Sci. USA.

