# Supplementary material for: Independent Evolutionary Lineages in a Globular Cactus Species Complex Reveals Hidden Diversity in a Central Chile Biodiversity Hotspot
Source: Genes (Basel). 2022 Jan 27;13(2):240. doi: 10.3390/genes13020240 (PMC8872226; doi:10.3390/genes13020240)
Supplement: Supplementary file 1 [file genes-13-00240-s001.zip › Table S1.pdf]

Table S1: Primers for the multiplex of microsatellite analysis

Name	Motive	PRIMER LEFT SEQUENCE	PRIMER RIGHT SEQUENCE	Ta (ºC)
PS5	CG	AACTAGGGAGTAGGAGTGTGTCAT	TGGGCTGCTAAAGGAAGAGA	61
PS9	GT	AAATCCCAAATCTGCGCAAC	ACGCCTGCTAAATTCTAGGGT	58
PC2	AG	TGTCGATTGGGCAAACATT	GCACTATGATCAGGTAACTGCC	58
PC11	TG	CAGCACAGTTAAGACTCAGGTCA	TAGACGGTCAAAGTGCTGCT	61
PM10	TC	GACTAGTTGGAGTCCGAAGCC	CCAGAAGTAGAGGAACCTACCC	61
PA12	TGC	GTGGGCCCTACAGGATGAT	CGGTAACTGATTCGGAGCAA	58
PM8	GT	CCTAATGCAATGTCGCTCCT	CACATGTGGTCCCACACAAT	58
PC10	TC	GTTGTTCTGGGTACGGTTGG	TTTGTTATGGCCGCTTGTTT	58
PM6	CT	ATGTCATGCACAATGCAGGT	AGTAATGGGCTGTCGATTGG	61
PC7	GA	TCATTGTGGAACAATAGAGGGA	CTTCTGCCAGACCCATTGAT	58
PM7	GA	CAACCGTGAATAATGGAGAGA	ATGAGCCCAGCCTACCCT	58
PA6	GA	CATGCATAGTGCCATGAAGC	TGCATTCAAGCACAACTTCC	58

Ta = annealing temperature
