# Supplementary figures and images for: Independent Evolutionary Lineages in a Globular Cactus Species Complex Reveals Hidden Diversity in a Central Chile Biodiversity Hotspot
Source: Genes (Basel). 2022 Jan 27;13(2):240. doi: 10.3390/genes13020240 (PMC8872226; doi:10.3390/genes13020240)

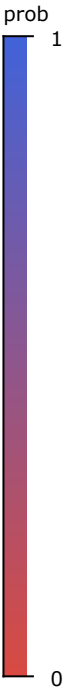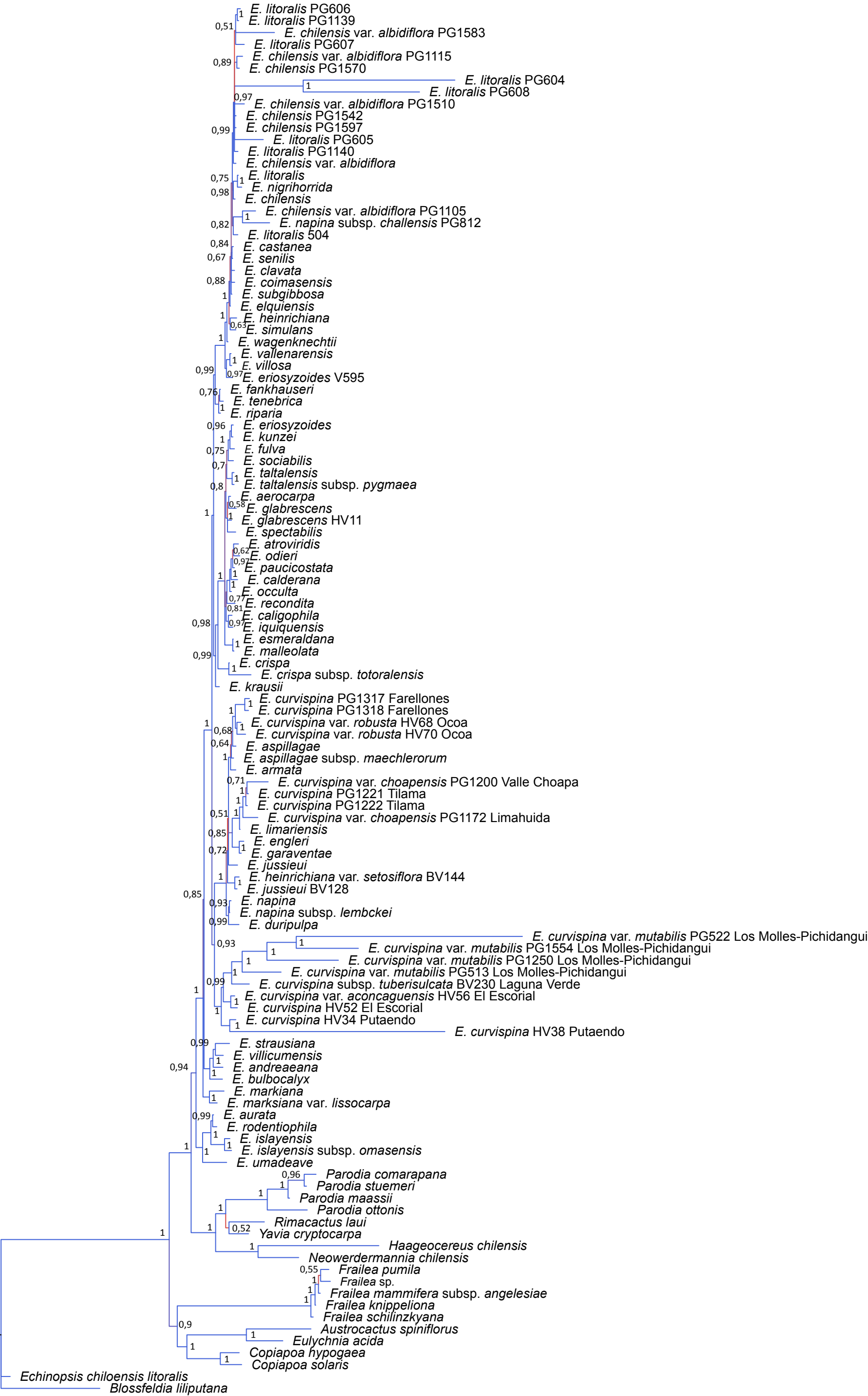

0.04

Supplement: Supplementary file 1 [file genes-13-00240-s001.zip › FigS1MatSuppl.pdf]

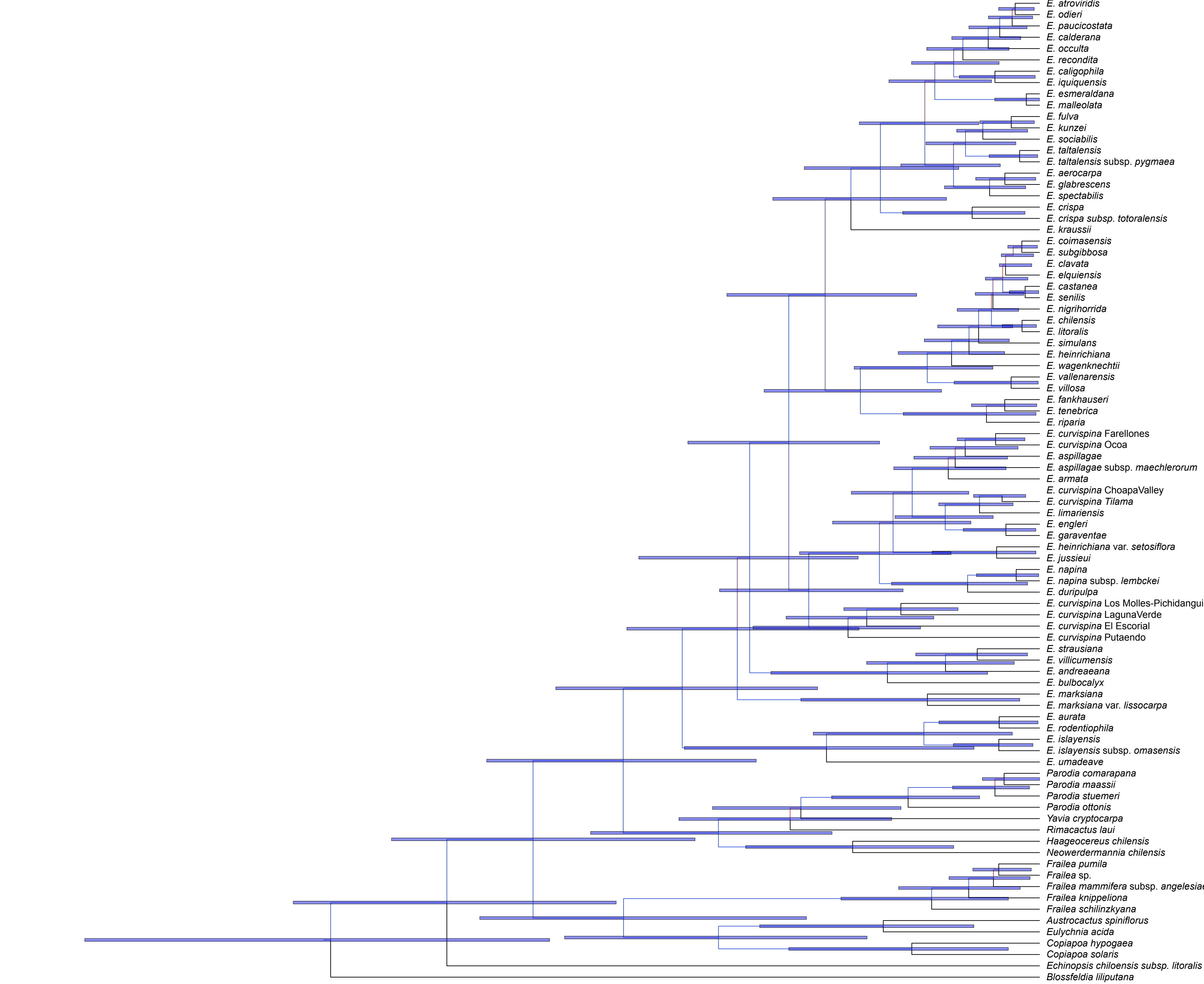

Supplement: Supplementary file 1 [file genes-13-00240-s001.zip › FigS2MatSuppl.pdf]

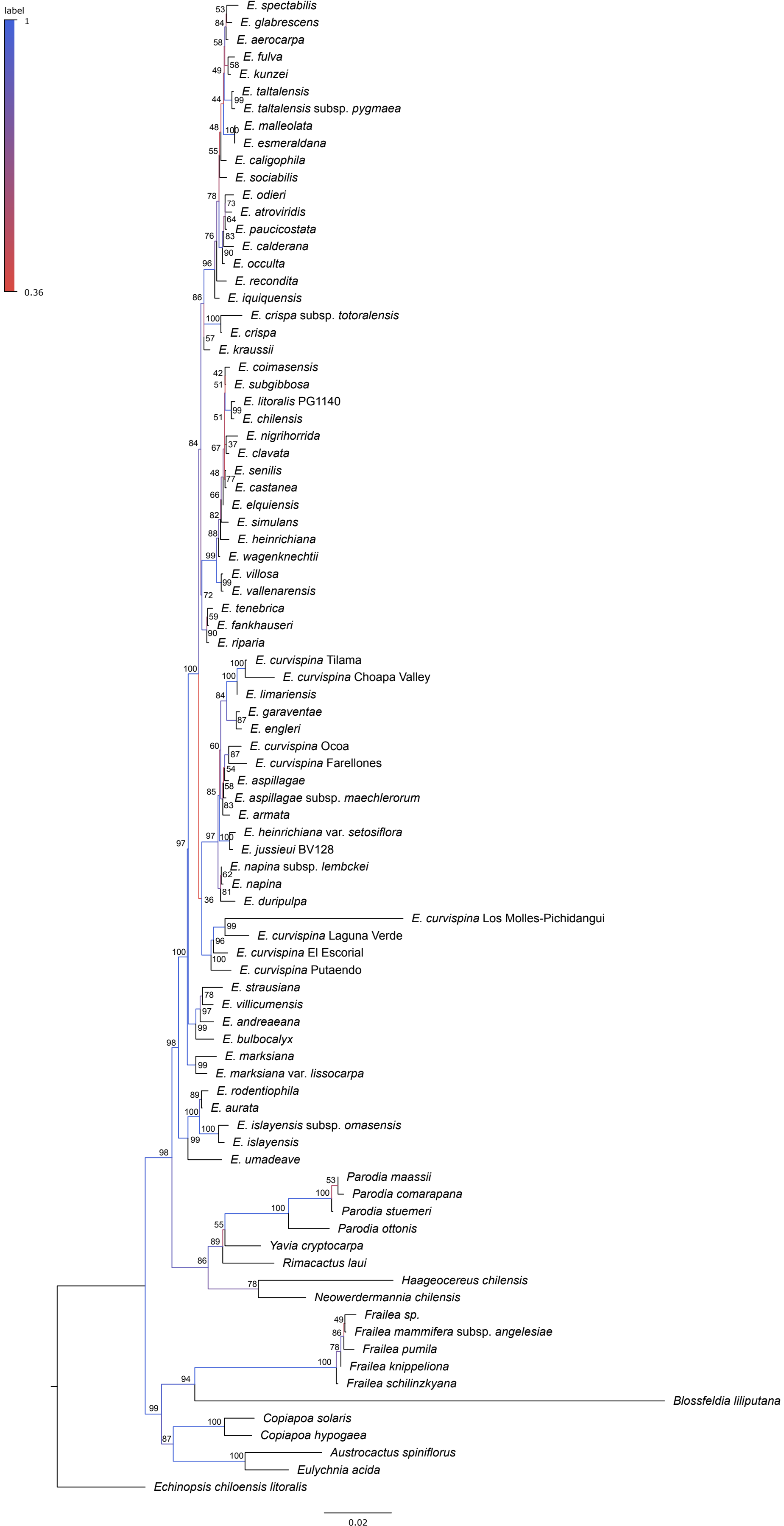

Supplement: Supplementary file 1 [file genes-13-00240-s001.zip › FigS3MatSuppl.pdf]

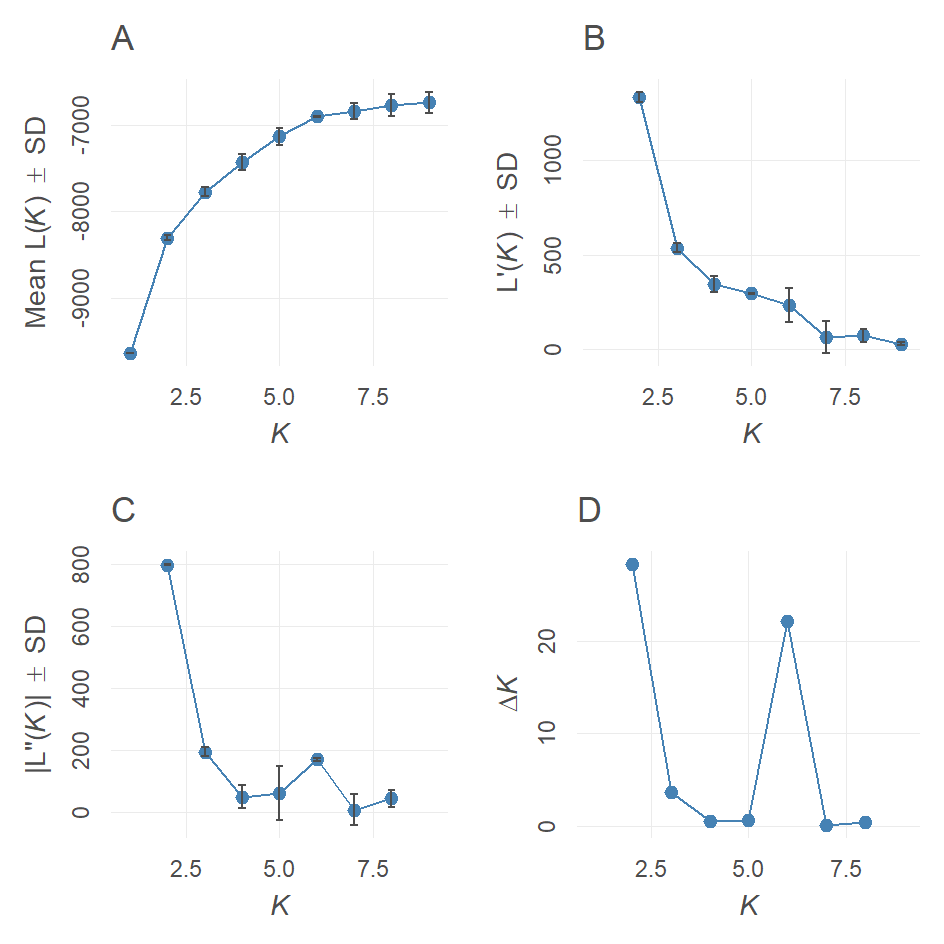

Supplement: Supplementary file 1 [file genes-13-00240-s001.zip › FigS4MatSuppl.png]
